# Choroidal Thickening During Young Adulthood and Baseline Choroidal Thickness Predicts Refractive Error Change

**DOI:** 10.1167/iovs.63.5.34

**Published:** 2022-05-26

**Authors:** Samantha Sze-Yee Lee, David Alonso-Caneiro, Gareth Lingham, Fred K. Chen, Paul G. Sanfilippo, Seyhan Yazar, David A. Mackey

**Affiliations:** 1 The University of Western Australia, Centre for Ophthalmology and Visual Science (incorporating the Lions Eye Institute), Perth, Western Australia, Australia; 2 Contact Lens and Visual Optic Laboratory, School of Optometry and Vision Science, Queensland University of Technology, Brisbane, Queensland, Australia; 3 Centre for Eye Research Ireland, School of Physics, Clinical and Optometric Sciences, Technological University Dublin, Ireland, Dublin, Ireland; 4 Department of Ophthalmology, Royal Perth Hospital, Perth, Western Australia, Australia; 5 Centre for Eye Research Australia, University of Melbourne, Royal Victorian Eye and Ear Hospital, East Melbourne, Victoria, Australia; 6 Garvan-Weizmann Centre for Cellular Genomics, Garvan Institute of Medical Research, Sydney, New South Wales, Australia; 7 School of Medicine, Menzies Research Institute Tasmania, University of Tasmania, Hobart, Tasmania, Australia

**Keywords:** choroidal thickness, myopia, axial length, young adults, the Raine Study

## Abstract

**Purpose:**

The purpose of this study was to explore the age-related change in choroidal thickness (ChT) and test the hypothesis that baseline ChT is predictive of refractive error change in healthy young adults.

**Methods:**

Participants underwent spectral-domain optical coherence tomography (SD-OCT) imaging and autorefraction at 20 (baseline) and 28 years old. The enhanced depth imaging mode on the SD-OCT was used to obtain images of the choroid. Scans were exported from the SD-OCT and analyzed with a custom software that automatically measures the central ChT. The longitudinal change in subfoveal ChT and association between baseline subfoveal ChT and 8-year change in refractive error (spherical equivalent) were determined using linear mixed models.

**Results:**

In total, 395 eyes of 198 participants (44% men; 18–22 years at baseline) were included. Over 8 years, mean spherical equivalent decreased by 0.25 diopters (D) and axial length increased by 0.09 mm. Subfoveal choroid thickened by 1.3 µm/year (95% confidence interval [CI] = 0.6–2.0), but this was reduced by 0.9 µm/year (95% CI = 1.6–0.2) for every 1 mm increase in axial length. For every 10 µm increase in baseline ChT, average annual change in spherical equivalent and axial length reduced by 0.006 D/year and 0.003 mm/year, respectively.

**Conclusions:**

In a community-based cohort of young adults, the choroid continued to change during early adulthood. Choroidal thickening was less in eyes that were longer at baseline, and the choroid thinned in eyes that showed myopia progression. The association between baseline ChT and longitudinal changes in spherical equivalent and axial length supports the hypothesis that ChT may be predictive of refractive error development and/or myopia progression.

Changes in choroidal thickness (ChT) have been implicated in several eye diseases. For example, thinner choroids have been noted in age-related macular degeneration,^1^ inherited retinal diseases,^2^ chronic posterior uveitis,^3^^–^^5^ and myopia,^6^^–^^12^ whereas thicker choroids have been linked with polypoidal choroidal vasculopathy^1^ and acute uveitis.^3^ Given that the choroid is the sole supplier of nutrients to the outer retina, changes in choroidal morphology may affect photoreceptor integrity and, therefore, visual acuity.

The relationship between thinner choroids and myopia has been well-described. It has been generally assumed that the choroid stretches and thins as a result of axial elongation in myopia. However, evidence from human^9^ and animal studies^13^ suggests that choroidal thinning precedes myopia development or progression. In a chick study where the eyes were allowed to develop naturally, Nickla and Totonelly^13^ demonstrated that eyes with thinner choroids elongated faster than those with thicker choroids. However, the authors did not find that this was the case in eyes that had experimentally induced myopia (e.g. by using lens-induced defocus or form deprivation). Fontaine et al.^9^ similarly noticed an inverse relationship between baseline ChT and rate of axial elongation over 15 months in 115 children, although it is unclear if the author's analysis corrected for age and sex. In young adults, exploration of the association between ChT and axial elongation has been limited, and thus there is a lack of understanding on how refractive error progression may be modulated by ChT (or vice versa) in this demographic.

In younger^6^^–^^12^^,^^14^^–^^16^ or older^17^^–^^20^ age groups, ChT and its variation with age have been profiled by several studies. Longitudinal^8^^,^^9^ and cross-sectional studies^6^^,^^7^^,^^14^^,^^15^^,^^21^ in Western populations have suggested that the ChT tends to increase during childhood, with a slower rate of thickening noted with faster axial elongation.^8^^,^^9^ On the other hand, studies in East Asia, which has a larger proportion of myopes, have argued that the ChT tends to decrease or does not vary with age during childhood.^11^^,^^12^ In adults above 50 years of age, cross-sectional studies have consistently demonstrated an inverse association between age and ChT.^17^^,^^18^^,^^22^ These reports suggest that there is a transition from choroidal thickening earlier in life to thinning during older age, and that this reversal occurs presumably sometime during young adulthood or middle age. An appreciation of the normal age-related changes in ChT throughout one's lifetime may be critical in our understanding of the choroid's role in development of or protection against age-related retinal diseases.

In this study, we examined the longitudinal change in ChT in healthy adults between 20 and 28 years of age and how this rate of change may be affected by refractive error changes. In view of previous observations^9^^,^^13^ that choroidal thinning precedes axial elongation, our second aim was to test the hypothesis that ChT at baseline (20 years old) predicts refractive error progression between 20 and 28 years.

## Methods

### Participants

This current investigation was conducted as part of the Raine Study.^23^ In brief, 2868 children born in the King Edward Memorial Hospital (Perth, Western Australia) in 1989 to 1992 were enrolled into the Raine Study, forming the original (Gen2) study cohort. At the 20- and 28-year examinations, Gen2 participants attended a comprehensive eye examination. All Raine Study examinations adhered to the Declaration of Helsinki and were approved by the University of Western Australia Human Research Ethics Committee. Prior to each examination, all participants provided informed written consent following an explanation of the nature of the study.

### Eye Examination

The 20- and 28-year examinations were conducted in 2010 to 2012 and 2018 to 2020, respectively, at the Lions Eye Institute (Perth, Western Australia) and have been described in detail previously.^24^^,^^25^ At both visits, participants underwent ocular biometry (IOLMaster version 5; Carl Zeiss Meditec AC, Jena, Germany) and conjunctival ultraviolet autofluorescence (CUVAF) photography (as an objective measure of ocular sun exposure),^26^ among other tests. Autorefraction (Nidek ARK-510A Autorefractometer; Nidek Co. Ltd., Tokyo, Japan) was performed at least 20 minutes after instillation of 1% tropicamide.

Refractive error was quantified in terms of spherical equivalent, calculated as the sum of the spherical value of the refraction plus half the cylindrical value. Myopia and high myopia were defined as per the International Myopia Institute guidelines.^27^ Eyes were determined to have a myopic or hyperopic shift if the change in spherical equivalent between the two examinations was ±0.50 diopters (D) or more.^28^ The 8-year change in axial length was expressed as a continuous variable and in tertiles.

### Choroidal Thickness Measurements

During the eye examination, participants underwent spectral-domain optical coherence tomography (SD-OCT) imaging (Spectralis HRA + OCT; Heidelberg Engineering, Heidelberg, Germany) with the Enhanced Depth Imaging (EDI) mode implemented to acquire images of the central choroid. SD-OCT was performed after dilation with tropicamide 1%. Prior to imaging, the corneal curvature for each eye was entered into the system as per the manufacturer's recommendation to correct for magnification effects. Two horizontal and 2 vertical 30 degrees single-line scans centered on the fovea were obtained, and an average of 100 frames for each scan was recorded. The same image acquisition protocol was applied at the 28-year examination, with the 20-year scans set as the reference on the SD-OCT.

EDI images were extracted from the SD-OCT and imported into a noncommercial custom software that uses machine-learning methods to automatically delineate the choroid (Supplementary Fig. S1).^29^^,^^30^ This method has been tested with SD-OCT EDI scans in a sample of participants with demographics similar to those of the current study, and shown to have high agreement with manual measurements of ChT.^30^ The program has a mean boundary position error of −0.19 and 1.25 pixels at the outer boundary retinal pigmented epithelium and chorioscleral interface, totaling to a mean ChT measurement error of 1.06 pixel, which corresponds to 4 µm.^29^ Each delineated image was checked by a trained observer who manually corrected the choroidal boundaries, if necessary, and the software then measured the ChT at 10 µm intervals. Tilted scans, usually due to long axial lengths, were corrected using the software, if required, prior to the image analysis. Non-EDI scans were not able to provide a clear view of the chorioscleral boundary^31^ and thus were not suitable for automated or manual image analysis. Given that the SD-OCT assumes a fixed axial length during imaging, we corrected for transverse magnification effects induced by differing axial lengths with a customized MATLAB script (Mathworks, Inc., Natick, MA, USA).^32^ From these corrected measurements, the ChT was determined at the nine Early Treatment of Diabetic Retinopathy (ETDRS) regions: subfoveally (0.5 mm radius around the foveal center), and at the superior, inferior, temporal, and nasal aspects of the inner macular ring (region between 0.5 and 1.5 mm radii around the foveal center), and outer macular ring (region between 1.5 and 3.0 mm radii around the foveal center; see Supplementary Fig. S1).

### Statistical Analyses

Statistical analyses were conducted using RStudio version 3.6.3 (The R Foundation for Statistical Programming, Vienna, Austria; https://www.r-project.org/) and significance was set at *P* < 0.05. Continuous measures were expressed as mean and standard deviation or median and interquartile range, as appropriate.

Linear mixed-effect models (LMMs) with random intercept and slope for participants to account for the within-participant correlation between the two eyes^33^ were used for all analyses. For the primary aim, the main outcome measure was the subfoveal ChT. Multivariable LMMs were generated to adjust for sex, ethnicity, and axial length, as well as the interaction effect between each of these variables and age on the ChT. For the purpose of statistical analysis, the age and axial lengths were centered at the means.

For the secondary aim, the outcome measures were the 8-year change in spherical equivalent and axial length, calculated as the difference in each of these measures between the 20- and 28-year visits. Subfoveal ChT at baseline was entered into the LMM as the independent measure of interest, with sex, ethnicity, axial length, and CUVAF area (as a measure of ocular sun exposure) entered as potential confounders.

Based on our previous observations of this study cohort,^32^^,^^34^ intraocular pressure, central corneal thickness, and time of OCT imaging were not associated with ChT and thus not included as covariates in the current analyses. Change in ChT was expressed in terms of micrometers per year (µm/year), as per our previous paper on 8-year refractive error change,^28^ and to allow comparison with studies of different durations.

## Results

At the 20-year examination, 1344 participants attended the eye examination. However, as the EDI mode on the SD-OCT was only available in mid-2011 — midway through the data collection phase — EDI scans were only performed for a subset of participants.^32^ At the 28-year examination, all 798 participants who attended the study visit had EDI scans obtained, but data collection ceased prematurely in early 2020 due to the coronavirus disease 2019 (COVID-19) pandemic. Thus, many of the participants who had ChT data at the 20-year examination were not able to attend the 28-year visit. After excluding erroneous or low-quality scans (SD-OCT signal-to-noise ratio <20)^35^ and eyes with possible pathology, as determined from the eye examination and using self-report, 385 eyes of 198 participants were available for analysis (see Supplementary Fig. S2 for detailed exclusion numbers).

At the baseline visit, there was no significant difference in sex, ethnicity, or prevalence of myopia at 20 years between participants with and without baseline ChT data (*P* > 0.05). However, participants who had baseline ChT measurements were, on average, 0.2 years younger than those who did not. This was because participants were contacted in birth order, thus, younger participants attended the 20-year examination later in the study when the EDI mode on the SD-OCT was available.

Among the 444 participants with baseline ChT data, there was no significant difference in age, sex, ethnicity, or prevalence of myopia or high myopia between the final sample included in the current analysis (*n* = 198) and those excluded (*P* > 0.05).

Table 1 shows the age and refractive error of the final study sample, which comprise 169 (84.5%) Caucasians, 9 (4.5%) East Asians, 3 (1.5%) South Asians, and 17 participants (9.5%) of mixed or other ethnicities. Eighty-eight (44%) participants were men. The mean duration between the 20- and 28-year examinations was 8.4 years (SD = 0.4 and range = 6.5–9.3).

**Table 1.** Characteristics of Included Sample at the 20-Year and 28-Year Examinations (*n* = 198)

Characteristic	20-Year	28-Year
Age, y	Range: 19.4 to 21.8 Mean = 19.9 (SD = 0.5)	Range: 27.1 to 29.3 Mean = 27.9 (SD = 0.3)
Axial length, mm	Median = 23.5 [IQR = 23.0 to 24.0]	Median = 23.6 [IQR = 23.1 to 24.2]
• 8-y change	Median = +0.09 [IQR = −0.01 to +0.22]	
Spherical equivalent (D)	Median = 0.38 [IQR = −0.25 to +0.63]	Median = 0.13 [IQR = −0.63 to +0.50]
• 8-y change	Median = −0.25 [IQR = −0.50 to +0.00]	
Myopes *n* (%)	48 (24.1)	63 (31.7)
High myopes *n* (%)	2 (1.0)	4 (2.0)

SD, standard deviation; IQR, interquartile range.

Participants were, on average, less hyperopic at 28 than at 20 years (both *P* < 0.001). Between the two examinations, an additional 15 and 2 participants developed myopia and high myopia, respectively (see Table 1). The majority of participants (68.1%) did not exhibit a shift in refractive error over the 8 years, whereas 27.2% and 4.7% had myopic and hyperopic shifts, respectively.

### Age Effect on Choroidal Thickness

On average, participants had thicker choroids at the 28-year compared to the 20-year visit. After adjusting for sex, ethnicity, and axial length, the subfoveal choroid thickened at an average rate of 1.3 µm/year (Table 2). As shown in Figure a and the Supplementary Table, the choroid thickened at the superior, inner nasal, and inner inferior regions by 1.1 to 1.8 µm/year. The outer temporal region, however, thinned by 1.2 µm/year, whereas the other regions showed no significant change with age (see Fig. a, Supplementary Table). Change in choroidal thickness over time did not significantly differ with sex or ethnicity (interaction terms *P* ≥ 0.07).

**Table 2.** Multivariable Analyses for Associations with Subfoveal Choroidal Thickness (µm)

Measure	Estimate [95% CI]	Statistical Outcome
*Main effects*		
Age, y	1.3 [0.6 to 2.0]	F_1,607_ = 13.8; *P* < 0.001^*^
Axial length, mm	−24.4 [−44.3 to −4.6]	F_1,754_ = 5.8; *P* = 0.016^*^
Age × axial length	−0.9 [−1.6 to −0.2]	F_1,587_ = 6.6; *P* = 0.010^*^
Male sex (ref = female)	26.9 [7.3 to 46.5]	F_1,193_ = 7.2; *O* = 0.008^*^
*Ethnicity (ref* = *Caucasians)*		F_1,334_ = 2.6; *P* = 0.42
• East Asians	−15.9 [−54.7 to 22.9]	
• South Asians	−32.7 [−63.3 to −2.1]	
• Other/mixed	−75.1 [−153.2 to 2.9]	

CI, confidence interval.

* Statistical significance at *P* < 0.05.

**Figure.**
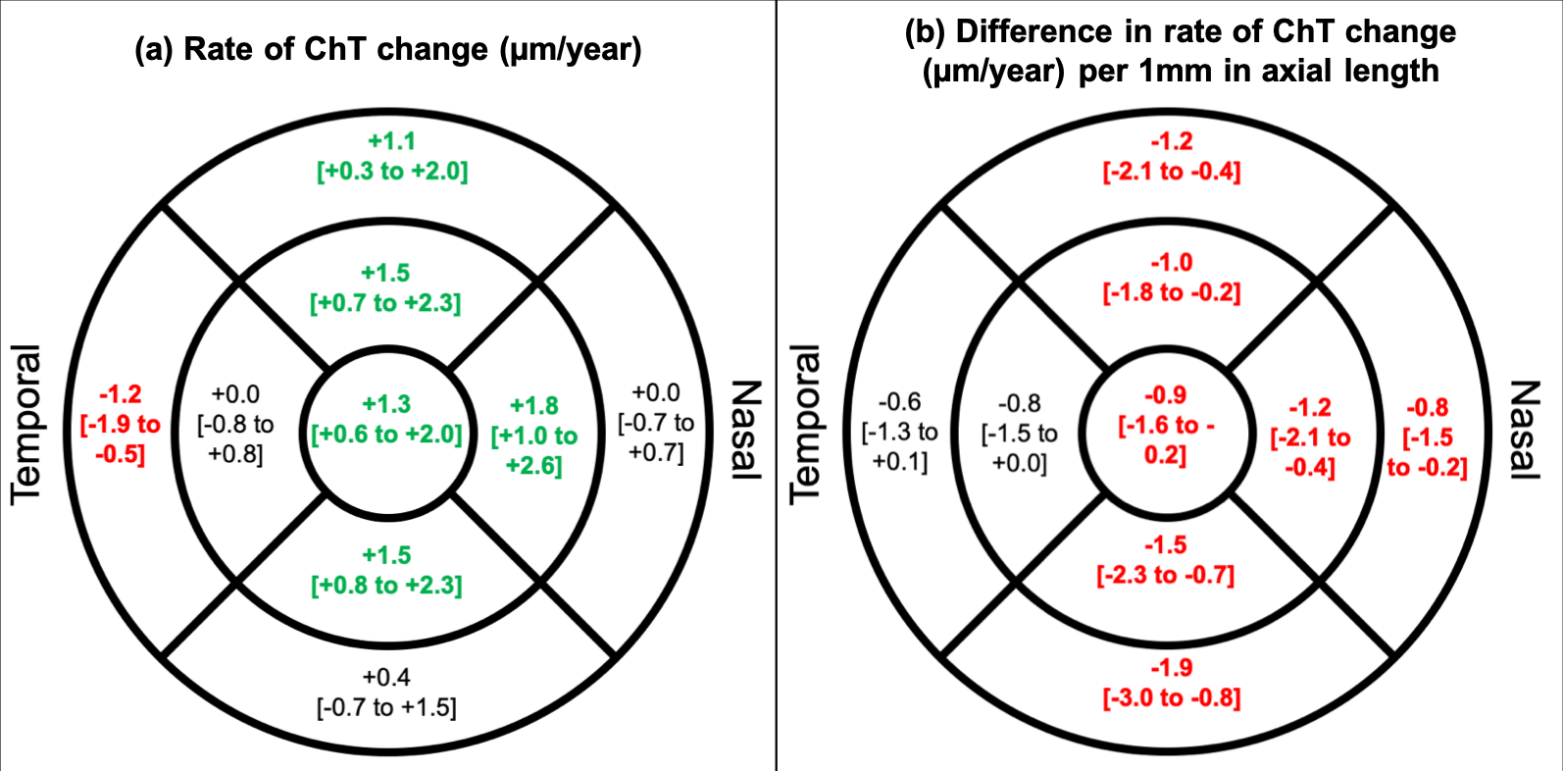
(**a**) Estimated change (and 95% confidence interval) in choroidal thickness (µm/year) at the 9 ETDRS regions. (**b**) Estimated difference (and 95% confidence interval) in rate of choroidal thickening (µm/year) per 1 mm increase in axial length. The nine regions shown are: subfovea (the innermost ring; 0.5 mm radius around the foveal center), and at the superior, inferior, temporal, and nasal aspects of the inner macular ring (between 0.5 and 1.5 mm radii around the foveal center), and outer macular ring (between 1.5 and 3.0 mm radii around the foveal center). Statistically significant changes (*P* < 0.05) shown in bold. Values in *green* represent a thickening, whereas those in *red* represents thinning or a decrease in rate of change.

### Longitudinal Change in Choroidal Thickness With Axial Elongation

Longitudinal choroidal thickening decreased with larger magnitudes of axial elongation and decreases in spherical equivalent (Supplementary Fig. S3). With each 1 mm increase in axial length, the amount of choroidal thickening decreased by 0.8 to 1.9 µm/year. This was noted at all but the inner and outer temporal ETDRS regions (see Fig. b, Supplementary Table).

As shown in Table 3, the 8-year subfoveal choroidal thickening was greatest in the group with the lowest tertile of 8-year axial elongation (least elongation). Similar trends were noted all other ETDRS regions, where there was a relative thinning or less thickening with higher tertile of axial elongation (*P* < 0.001 to 0.011; data not shown).

**Table 3.** Eight-Year Change in Subfoveal ChT in Eyes With Axial Elongation (in Tertiles) and Refractive Error Shift

8-y Change	8-y Change in ChT, µm	Statistical Difference^*^	*P* Value
*Axial elongation, mm*			
• Tertile 1: <0.023 (*n* = 128 eyes)	Median = +30.4[IQR = +0.5 to +59.6]	Reference	Reference
• Tertile 2: 0.023–0.170 (*n* = 127 eyes)	Median = +7.0[IQR = −15.5 to 34.0]	Estimate = −22.4[95% CI = −35.8 to −8.9]	0.001^‡^
• Tertile 3: >0.170 (*n* = 130 eyes)	Median = −22.5[IQR = −63.6 to +11.8]	Estimate = −56.3[95% CI = −71.2 to −41.4]	<0.001^‡^
*Refractive error shift* ^†^			
• None (*n* = 263 eyes)	Median = +10.9[IQR = −12.3 to +41.6]	Reference	Reference
• Hyperopic (*n* = 18 eyes)	Median = +40.9[IQR = −2.8 to +71.2]	Estimate = 13.3[95% CI = −7.7 to +34.3]	0.22
• Myopic (*n* = 104 eyes)	Median = −19.1^‡^[IQR = −61.3 to −25.3]	Estimate = −20.0[95% CI = −31.2 to −8.7]	0.005^‡^

* Axial length and refractive error shift included as categorical variables in separate linear mixed models, with subfoveal choroidal thickness as the outcome measure and adjusted for sex and ethnicity.

† Refractive error shift determined as a change in spherical equivalent of 0.50 D over the 8-year period.

‡ Statistically significant at *P* < 0.05.

ChT, choroidal thickness; CI, confidence interval; IQR, interquartile range.

In eyes that exhibited a myopic shift, the choroid thinned over 8 years, in contrast to the choroidal thickening observed from those with hyperopic shift or no refractive error change. There was no significant difference in choroidal thickening between eyes with hyperopic shift and eyes with no refractive error change (see Table 3), although this lack of statistical significance may be due to the small number of eyes with a hyperopic shift.

### Choroidal Thickness at Baseline and Change in Refractive Error

As shown in Table 4, having thicker choroids at baseline was associated with a smaller change in spherical equivalent. For every 10 µm increase in subfoveal ChT at baseline, the change in spherical equivalent and axial elongation were decreased by 0.006 D/year and 0.0003 mm/year (less myopic), respectively. To further explore whether the association between baseline ChT and 8-year change in these measures is affected by the presence of myopia at baseline, we included a baseline myopia × baseline ChT interaction term in separate models. This interaction effect was not statistically significant (*P* = 0.21 for spherical equivalent and *P* = 0.09 for axial length), suggesting that the presence of myopia at baseline did not affect the relationship between baseline ChT and 8-year change in spherical equivalent or axial length.

**Table 4.** Associations of 8-Year Change in Spherical Equivalent and Axial Length

Measure	Estimate [95% CI]	Statistical Outcome
**Spherical equivalent**		
Male sex (ref = female)	+0.21 [+0.06 to +0.37]	F_1,189_ = 7.4; *P* = 0.007^*^
*Ethnicity (ref* = *Caucasians)*		F_1,189_ = 2.0; *P* = 0.07
• East Asians	+0.36 [−0.03 to +0.74]	
• South Asians	+0.42 [−0.19 to +1.03]	
• Other/mixed	−0.11 [−0.38 to +0.16]	
CUVAF (per 10 mm^2^)	+0.02 [−0.01 to +0.04]	F_1,190_ = 2.3; *P* = 0.14
Subfoveal ChT (per 10 µm) at baseline	+0.006 [+0.001 to +0.013]	F_1,370_ = 4.1; *P* = 0.047^*^
**Axial length**		
Male sex (ref = female)	−0.06 [−0.12 to +0.01]	F_1,189_ = 3.2 *P* = 0.07
*Ethnicity (ref* = *Caucasians)*		F_1,189_ = 1.9; *P* = 0.10
• East Asians	+0.14 [−0.02 to +0.30]	
• South Asians	−0.20 [−0.46 to +0.06]	
• Other/mixed	+0.04 [−0.08 to +0.15]	
CUVAF, per 10 mm^2^	−0.01 [−0.02 to +0.00]	F_1,190_ = 3.1; *P* = 0.08
Subfoveal ChT at baseline, per 10 µm	−0.003 [−0.006 to −0.000]	F_1,348_ = 4.2; *P* = 0.041^*^

Subfoveal ChT, choroidal thickness at central 0.5 mm radius around the fovea; CI, confidence interval; CUVAF, conjunctival ultraviolet autofluorescence.

* Statistical significance at *P* < 0.05.

## Discussion

In this 8-year longitudinal study of community-based young adults, we demonstrated that, on average, the choroid continued to thicken during the third decade of life, although in eyes with a myopic shift (≤−0.50 D) or axial elongation, the choroid thinned over the 8 years. This is in contrast to previous studies in adults that have suggested otherwise — that the ChT decreases or does not change with age.^22^^,^^36^^–^^42^ It is worth noting that these previous studies were cross-sectional in nature, and thus the findings may have been affected by generational changes in environmental factors, rather than a true age effect, as has been the case for refractive errors.^43^ For example, decreasing rates of smoking in recent decades^44^^,^^45^ may contribute to the relatively thicker choroids in younger generations (and thinner choroids in the older generations), resulting in an apparent negative relationship between age and ChT in cross-sectional observations. Moreover, many previous studies only looked at middle-aged adults or older,^46^^,^^47^ when a true age-related choroidal thinning may have occurred.

In other studies where young adults were nested within a population with a wide age range, the statistical methods used only considered a linear relationship and thus could not detect a differential rate of ChT change with age.^32^^,^^36^^,^^38^^–^^40^^,^^48^ For example, in participants 12 to 80 years of age, Akhtar et al.^36^ reported that the subfoveal choroid in those aged 18 to 30 years were thicker than those younger (12–18 years) or older (above 30 years), yet concluded that ChT decreases by approximately 2.5 µm/year. Similar findings where authors have not considered a nonlinear relationship between ChT and age are seen in several other studies.^22^^,^^38^^–^^40^

Age-related choroidal thickening, as observed in our cohort of young adults, has been described in children. Xiong et al.^12^ reported that the choroid thickened by 3.7 µm/year in emmetropic children 6 to 19 years old. In a 1-year longitudinal study by the same authors,^48^ the ChT was reported to show no significant change between 6 and 9 years old, but then thicken by 9 µm/year between 10 and 13 years old in children with no myopic shift. In concordance with Xiong et al.'s latter finding, Read et al.'s longitudinal study^8^ noted an 8 µm/year choroidal thickening in 10- to 15-year-old children. In the Copenhagen Child Cohort 2000,^49^ faster rates of choroidal thickening over 5 years were noted in girls with larger growth spurts and more advanced pubertal development. However, this association was not noted in boys, which may be due to boys experiencing pubertal development at a later age than girls.

Thickening of the choroid is believed to be part of the ocular developmental process after birth and during childhood.^50^ Ocular growth and development, including emmetropization, occurs predominantly during early childhood and at decreasing rates as a child grows older. However, it is not clear when such developmental processes stop. Our recent study^28^ demonstrated that axial elongation and myopic shifts in refractive error, which occurs during the emmetropization process, continued in about one-third of young adults in a representative Australian population. This, together with our current findings on choroidal thickening in this cohort, suggest that ocular development continues during early adulthood, albeit less than during childhood. The attenuated choroidal thickening in the current cohort may also represent a gradual transitioning from choroidal thickening to thinning, as observed during older age.^17^^,^^18^^,^^22^

We additionally observed decreased choroidal thickening with faster axial elongation or with myopic shift over the 8 years, although there was considerable variation between ETDRS regions. We found that with longer axial lengths, choroidal thinning was most prominent at the nasal and inferior regions, in agreement with previous reports in children.^7^^,^^51^ Although choroidal thinning may be a result of eye elongation, our findings also suggest that choroidal thinning may precede a myopic shift or axial elongation. Thus, a thinner choroid nasally or at the posterior pole may simply be a precursor to a myopic shift during young adulthood. Researchers have posited that ChT modulates eye growth in response to optical defocus.^52^ Given that ChT can change more readily than the eye can elongate, Nickla and Wallman^52^ postulated that transient but repeated episodes of choroidal thinning induced by brief periods of hyperopic defocus (a proposed mechanism of myopigenesis) may drive ocular elongation via a mechanical or molecular mechanism. One of the mechanisms suggested by the authors^52^ for this effect was that thicker choroids pose a greater physical barrier for molecular signals and growth factors traversing from the retina to the sclera. Our findings, along with those of previous studies on children^9^ and chicks,^13^ support the notion that choroidal thinning precedes myopic shift, and thus ChT may play a role in development of refractive error and emmetropization. Given the small magnitude of longitudinal change in refractive error and axial length in our participant cohort, further studies comprising children with larger shifts in refractive error are required to confirm this observation and hypothesis. This could have important implications in myopia control research. For example, the low-concentration atropine for myopia progression study^53^ recently showed that atropine eyedrops have a dose-dependent long-term choroidal thickening effect, which the authors suggested could help to slow the rate of axial elongation.

The main strengths of the study were the longitudinal nature of our study and that our participants from the Raine Study have been shown to be generally representative of the general Western Australia population of similar-aged adults.^54^ However, approximately 85% of participants were not included in this study due to the unavailability of the EDI modes on the SD-OCT early in the study and during the COVID-19 pandemic. We nevertheless managed to find a significant age-related change in ChT with the included study sample. Moreover, our current sample size remains larger than or similar to those of many previous longitudinal^8^^,^^9^^,^^11^^,^^55^ or cross-sectional^7^^,^^10^^,^^15^^,^^17^^,^^20^^,^^21^ studies (approximately 100–200) that have described age-related variations in the choroidal thickness. Although there was a statistically significant age difference between participants included and excluded from the analysis, the age difference was small (approximately 0.2 years or 2.4 months). Another limitation is that the choroid undergoes diurnal variation but we did not control for the timing of visits in this study. Furthermore, we did not control for caffeine intake, smoking, or exercise, all of which could have transient and long-term effects on the choroidal thickness.^56^^–^^58^ Given that the total number of participants who attended the eye examination was relatively large (*n* = 798), we were unable to control for the time of day participants visited or ask about their activities during the day prior to the eye examination. In our previous analyses,^19^^,^^21^ we addressed this by including the time of OCT scan in the statistical models; however, we did not find that it improved model fit (based on the relevant information criterion) or changed the statistical outcomes, and thus did not do this in the current study. Another potential limitation was that the eye examination and SD-OCT scans were conducted 8 years apart. We were thus unable to ascertain any differential rate of choroidal thickening over the 8 years; for example, early 20s versus mid- or late 20s. Finally, SD-OCT scans were conducted after mydriatic instillation, which may have resulted in slight choroidal thickening, although it has been argued that tropicamide does not significantly alter ChT.^59^ Moreover, mydriasis allowed for the acquisition of higher-quality images and because this was done at both visits (20- and 28-years), it was unlikely to have a significant effect on the amount of longitudinal change.

In our community-based cohort of young healthy adults, the choroid continued to thicken during the third decade of life, albeit less than previously observed in children. The amount of thickening was inversely associated with axial length. Our findings are compatible with the hypothesis that thinner choroids precede a myopic shift; however, further research – in children and in young adults – are warranted to support or refute this finding. As we continue to follow this cohort of participants, we will aim to determine the age at which the choroid starts to thin and investigate how this reversal in ChT changes is affected by the type of refractive errors and/or ocular pathology.

## Supplementary Material

Supplement 1

Supplement 2
