# Supplementary material for: Choroidal Thickening During Young Adulthood and Baseline Choroidal Thickness Predicts Refractive Error Change
Source: Invest Ophthalmol Vis Sci. 2022 May 26;63(5):34. doi: 10.1167/iovs.63.5.34 (PMC9150825; doi:10.1167/iovs.63.5.34)
Supplement: Supplement 1 [file iovs-63-5-34_s001.pdf]

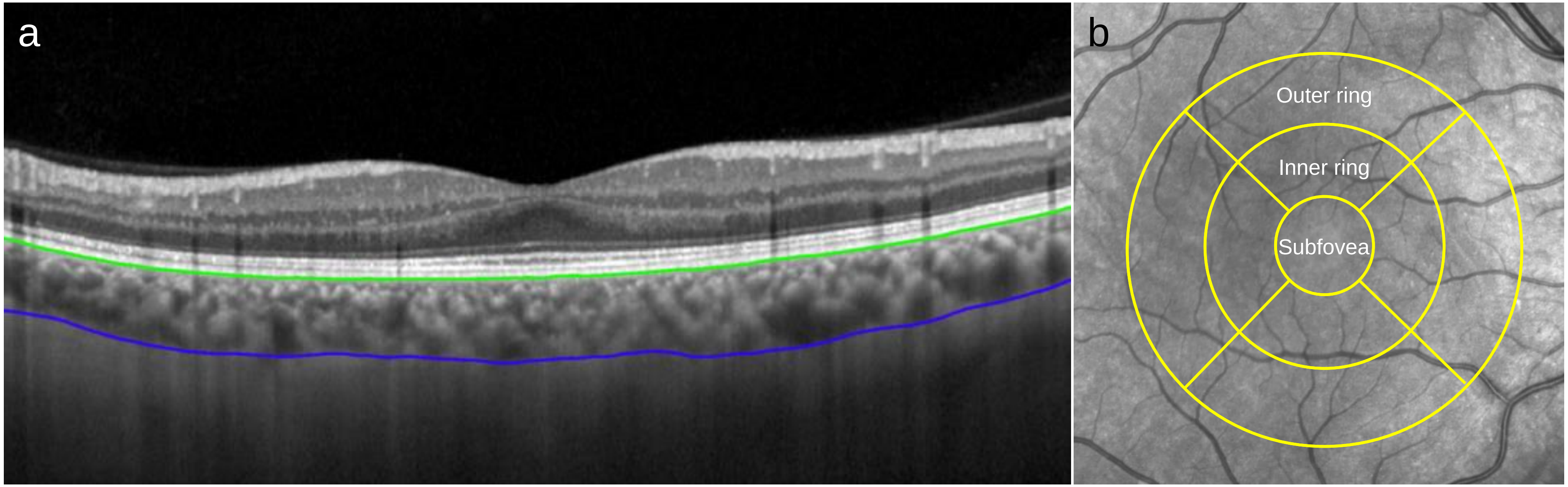

Supplementary Figure 1. *(a) Segmentation of the choroidal layer, with the green line delineating the outer boundary of the retinal pigmented epithelium and the blue line delineating the chorioscleral interface. (b) Choroidal thickness measured at the 9 Early Treatment of Diabetic Retinopathy (ETDRS) regions, comprising the subfovea (0.5mm radius around the fovea), as well as the superior, inferior, temporal, and nasal quadrants at the inner (between 0.5 and 1.5mm radii around the fovea) and outer (between 1.5 and 3.0mm radii around the fovea) macular rings*

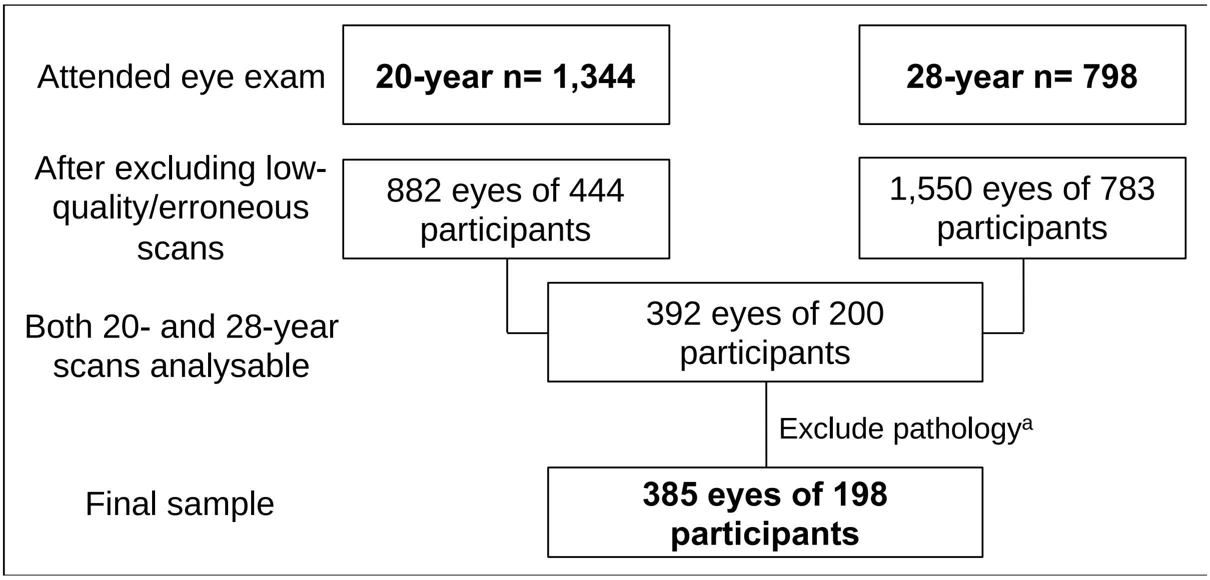

***Supplementary Figure 2**. Study sample. [a] Excluded: 2 eyes of 1 participant with resolved acute posterior multifocal placoid pigment epitheliopathy; 1 eye of 1 participant with resolved acute macular neuroretinopathy; 1 eye of 1 participant with central serous chorioretinopathy; 1 eye of 1 participant with retinal pigment epithelitis; and 2 eyes of 1 participant who was taking hydroxychloroquine.*

**Sample size was small as the enhanced depth imaging mode on the spectral domain optical coherence tomography was only available midway through the 20-year follow-up data collection phase and many participants who had choroidal thickness at the baseline were unable to return for the 28-year follow-up due to the COVID-19 pandemic*

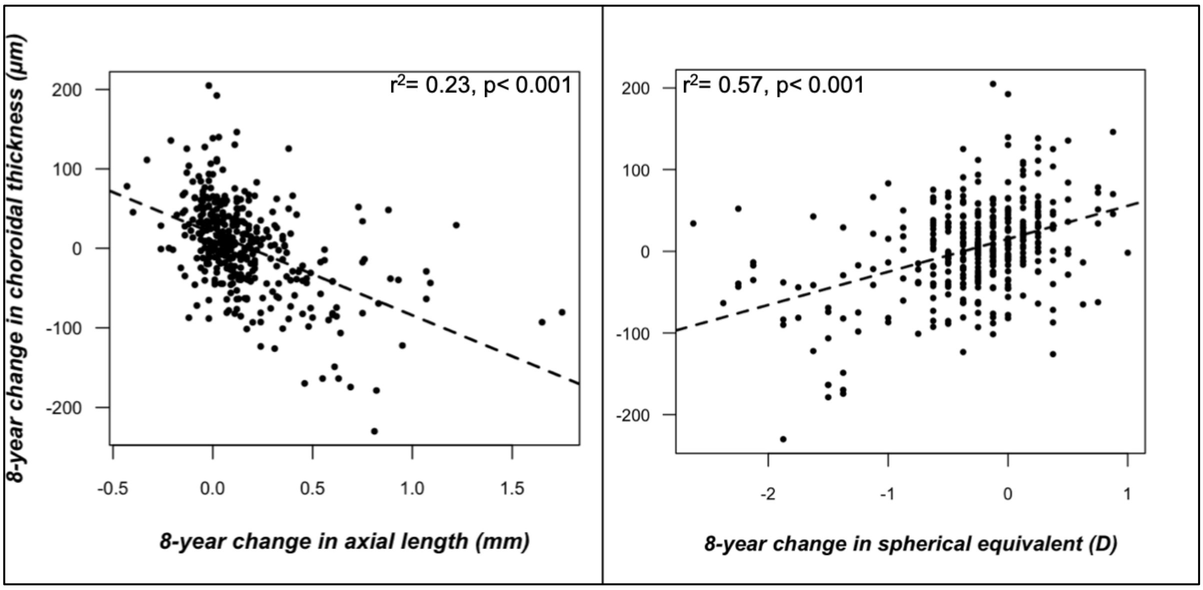

Supplementary Figure 3*. Longitudinal change in choroidal thickness as a function of change in axial length (left) and spherical equivalent (right)*
