# Supplementary material for: Choroidal Thickening During Young Adulthood and Baseline Choroidal Thickness Predicts Refractive Error Change
Source: Invest Ophthalmol Vis Sci. 2022 May 26;63(5):34. doi: 10.1167/iovs.63.5.34 (PMC9150825; doi:10.1167/iovs.63.5.34)
Supplement: Supplement 2 [file iovs-63-5-34_s002.pdf]

Supplementary Table. Change in choroidal thickness with age and axial length at the 8 non-central Early Treatment of Diabetic Retinopathy Study regions[a]

Measure	Estimate [95%CI]	Statistical outcome[b]
**Region: Inner superior**		
Age (year)	1.5 [0.7 to 2.3]	$F_{1,557}$= 12.6; p< 0.001**
Axial length (mm)	-16.6 [-38.6 to 5.4]	$F_{1,701}$= 2.2; p= 0.14
Age × axial length	-1.0 [-1.8 to -0.2]	$F_{1,540}$= 6.5; p= 0.011*
**Region: Inner inferior**		
Age (year)	1.5 [0.8 to 2.3]	$F_{1,559}$= 15.0; p< 0.001**
Axial length (mm)	-8.4 [-29.8 to 13.0]	$F_{1,703}$= 0.6; p= 0.44
Age × axial length	-1.5 [-2.3 to -0.7]	$F_{1,540}$= 14.8; p< 0.001**
**Region: Inner temporal**		
Age (year)	0.0 [-0.8 to 0.8]	$F_{1,566}$= 0.0; p= 0.94
Axial length (mm)	-18.8 [-40.3 to 2.8]	$F_{1,715}$= 2.9; p= 0.09
Age × axial length	-0.8 [-1.5 to 0.0]	$F_{1,553}$= 3.8; p= 0.05
**Region: Inner nasal**		
Age (year)	1.8 [1.0 to 2.6]	$F_{1,567}$= 19.7; p< 0.001**
Axial length (mm)	-16.2 [-39.3 to 6.9]	$F_{1,717}$= 1.9; p= 0.17
Age × axial length	-1.2 [-2.0 to -0.4]	$F_{1,553}$= 8.9; p= 0.003**
**Region: Outer superior**		
Age (year)	1.1 [0.3 to 2.0]	$F_{1,552}$= 7.0; p= 0.008**
Axial length (mm)	-9.6 [-32.5 to 13.2]	$F_{1,694}$= 0.7; p= 0.41
Age × axial length	-1.2 [-2.1 to -0.4]	$F_{1,537}$= 8.3; p= 0.004**
**Region: Outer inferior**		
Age (year)	0.4 [-0.7 to 1.5]	$F_{1,552}$= 0.5; p= 0.46
Axial length (mm)	9.5 [-18.4 to 37.3]	$F_{1,674}$= 0.4; p= 0.51
Age × axial length	-1.9 [-3.0 to -0.8]	$F_{1,543}$= 11.9; p= 0.001**
**Region: Outer temporal**		
Age (year)	-1.2 [-1.9 to -0.5]	$F_{1,565}$= 11.3; p= 0.001**
Axial length (mm)	-18.6 [-38.4 to 1.2]	$F_{1,716}$= 3.4; p= 0.07
Age × axial length	-0.6 [-1.3 to 0.1]	$F_{1,552}$= 2.9; p= 0.09
**Region: Outer nasal**		
Age (year)	0.0 [-0.7 to 0.7]	$F_{1,568}$= 0.0; p= 0.97

Axial length (mm)	-21.0 [-41.1 to -0.8]	$F_{1,718}$= 4.2; p= 0.042*
Age × axial length	-0.8 [-1.5 to -0.2]	$F_{1,550}$= 6.0; p= 0.015*

[a] Inner rings: between 0.5 and 1.5mm radius around the fovea, outer rings: between 1.5 and 3.0mm radius around the fovea; [b] corrected for sex and ethnicity.

CI= confidence interval

Statistical significance at *p< 0.05 and **< 0.01
